# P-1870. Environmental, Time, and Cost Benefits of Consolidated Subspecialty Care in a Multidisciplinary Pediatric Long COVID Clinic

**DOI:** 10.1093/ofid/ofaf695.2039

**Published:** 2026-01-11

**Authors:** Shreya Doshi, Erin McLaughlin, Madison Nemshick, Emily Ansusinha, Alexandra B Yonts

**Affiliations:** Children's National Health System, Washington DC, DC; George Washington University, Washington, District of Columbia; The George Washington University School of Medicine and Health Sciences, Washington, District of Columbia; Children's National Hospital, Washington, District of Columbia; Children's National Hospital/ George Washington University, Washington, District of Columbia

## Abstract

**Background:**

Multidisciplinary clinics are increasingly used to manage patients with complex medical conditions. Previous literature has demonstrated improved outcomes, patient satisfaction and clinician efficiency with multidisciplinary care. Pediatric Long COVID (LC) is a systemic condition defined as ≥ 1 symptom that begins ≤ 3 months after SARS-CoV-2 infection, lasts ≥ 2 months and impairs daily life. Children with LC require evaluation by multiple pediatric subspecialists. When scheduled separately, these visits are burdensome on families and generate excess greenhouse-gas (GHG) emissions. Our institution hosts a dedicated pediatric multidisciplinary LC clinic. We quantified the environmental and societal benefits of consolidating multiple consultations into a single-day multidisciplinary visit.

**Figure:**
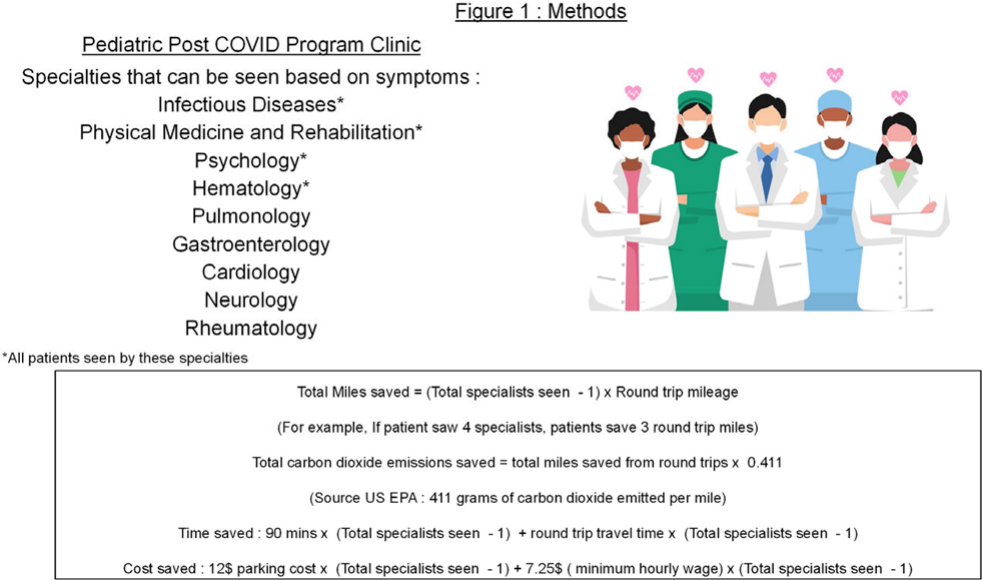

**Figure:**
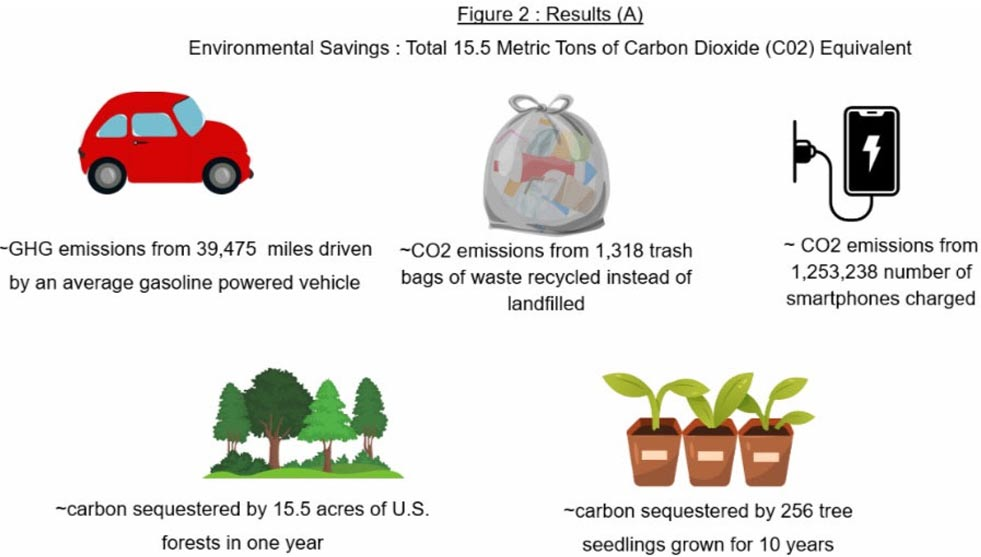

**Methods:**

Addresses for all local patients seen in the Post COVID Program Clinic at Children’s National Hospital from 5/2021-11/2024 were extracted from the electronic medical record and geocoded. One-way distance and travel time were calculated via Google Maps and doubled to estimate round-trip values. Assumptions included travel by private vehicle and that each subspecialist would have required a separate visit in a traditional model. The formulas and calculations used to determine savings are included in Figure 1. EPA “equivalency” conversions (miles driven, trash bags recycled, smartphones charged, tree-seedling/forest sequestration) were determined using the EPA calculator.

**Figure:**
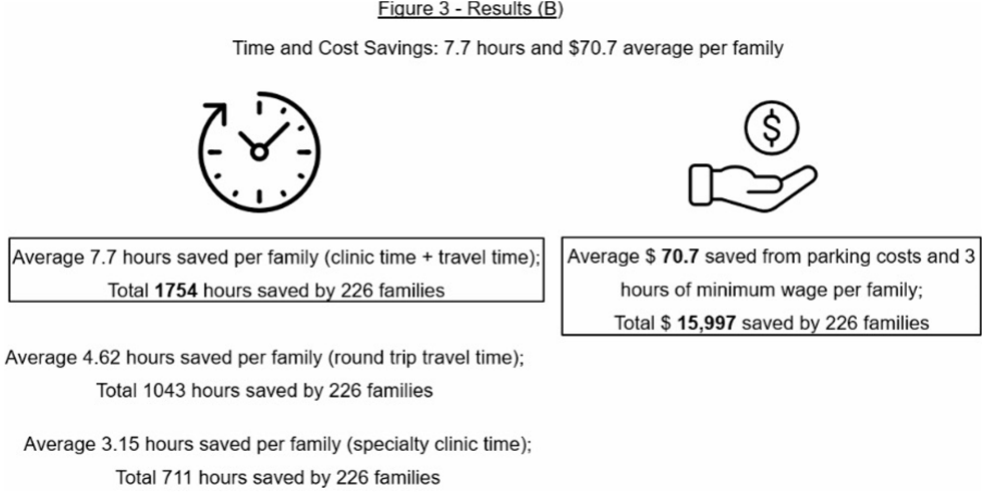

**Figure:**
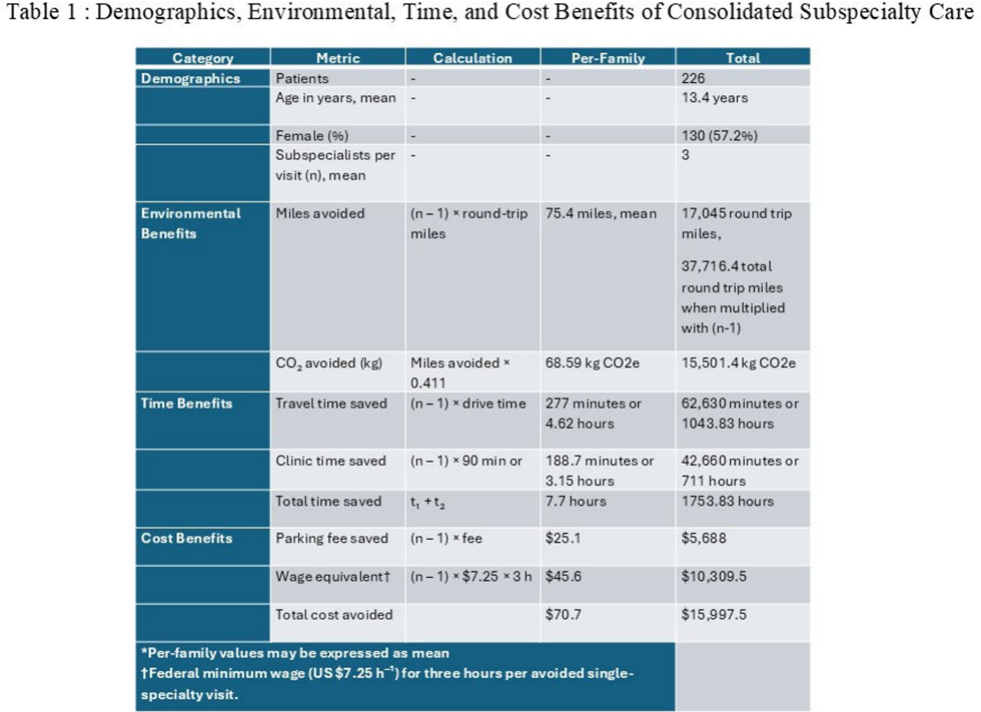

**Results:**

Among 226 pediatric patients, a median of 3 subspecialists were seen per visit. Consolidation of visits via the multidisciplinary clinic prevented 17,045 total road-miles and 15,501 kg CO₂ emissions (Table 1). Families cumulatively saved a total of 1,754 hours and at least $15,997. Figure 2 and Figure 3 illustrate environmental and cost/time savings.

**Conclusion:**

A single-day multidisciplinary pediatric Long COVID clinic—one of few nationwide—substantially reduces travel-related GHG emissions, time away from work/school, and out-of-pocket expenses. This consolidated model is readily transferrable to other complex pediatric conditions. The environmental benefits of telemedicine have been documented, but this is the first study of a multidisciplinary clinic.

**Disclosures:**

Alexandra B. Yonts, MD, Pfizer: Grant/Research Support

